# An Operation for Imperforate Anus

**Published:** 1882-05

**Authors:** R. Y. Rudicil

**Affiliations:** Summerville, Ga.


### AN OPERATION FOR IMPERFORATE ANUS.

By R. Y. RUDICIL, M.D., Summerville, Ga.

On the 22d of February, 1882, Mrs. L. was delivered of a male child, which seemed to be well developed and healthy, and of about nine pounds weight. The labor was conducted by a midwife. On the second day after birth, the child being attended by its grandmother, it was ascertained by her that the child had no anus. On the evening of the same day—that is, two days subsequent to its birth—I was called to see it; and finding upon examination that there was no anus, made preparations for an operation, which I performed the following morning, assisted by my partner, Dr. J. M. F. Myers, and Dr. L. C. Calhoun, of Summerville.

As there was no pouting or bulging of the tissues, we decided to make an incision a half-inch in length in the ordinary position of the anus, and carry the incision upward until the gut was reached, or until met by hemorrhage that would forbid further proceeding. When the incision was carried two inches in depth and no indication had appeared of the gut having been reached, we deemed it advisable to desist from further operation. Believing now that the rectum was absent or terminated in some other locality, and not feeling that we had accomplished any benefit by the operation, we decided to introduce no tent, knowing that relief for the child was only in death.

There being no prominence in the locality of the sigmoid flexure of the colon, nor indications of tympany on percussion, we considered it inexpedient to resort to operative measures in any of the localities suggested by writers on surgery, notwithstanding the parents of the child gave full permission to submit it to any operation we might think offered most favorable chances for relief. The child was left in this condition with the statement to the parents that death was inevitable.

I saw the child on the second day after the operation, and, upon examination of the incision, saw meconium pass in small quantity; found that gas passed by the incision; and also learned from its grandmother that gas and meconium had passed from the penis. Being now satisfied that the bowel by at least a small opening communicated with the bladder, and being furthermore satisfied by the discharges from the incision, that the bowel had been reached by the knife, but not having made an aperture sufficiently large for free exit of contents, we determined to perform a second operation and enlarge the opening into the bowel. This was done by carrying the incision one-half inch deeper, directing the point of the knife slightly in direction of the sacrum, from which opening discharges passed in small quantity for two or three days and then ceased, notwithstanding the wound was kept open by tents for several days. After the third day, there being no discharge from the wound, it was deemed useless to continue the tents longer. The incision was left to heal, and the child to die, as we considered the case utterly hopeless under any treatment.

Fecal matter or meconium continued to pass by the penis in very small quantities up to the 20th day, when death ended the child's suffering. Being allowed the privilege of an autopsy, Dr. J. M. F. Myers, in the presence of Drs. J. C. Calhoun of Summerville, J. J. Marsh of Subligna, J. B. S. Holmes of Rome, and myself, proceeded to open the abdomen by an incision from the umbilicus down the median line to the symphisis pubis, and a crucial incision extending from one process of the ilium to the other. No abdominal viscera were examined except the intestines and bladder. The whole cavity showed considerable peritoneal inflammation which was especially marked along the whole of the large bowel from the cæcum to the rectum. Complete and extensive adhesion to the abdominal walls from the arch of the colon to the terminus of the rectum was found to exist.

The rectum was found along the pelvic walls in the usual position, but extending down into Douglas' cul de sac, where it made a sharp curve upon itself, and, after extending two inches upwards, made another curve downwards and attached itself into the bladder just behind the prostate gland. A small aperture was found to open into the bladder, just large enough to admit an ordinary-sized probe, through which meconium, as before stated, had passed into the bladder and out through the penis.

The adhesions at the sigmoid flexure constricted the calibre of the bowel so much at this point that, together with the distended bladder which pressed the upper and unnatural curve of the rectum back upon itself or some other portion of the bowel, as it passed down the pelvic wall, it almost entirely prevented the colon from sending its contents below the point of occlusion. The whole of the large bowel above this point was found to contain a considerable amount of fecal matter, while that part of the rectum below the points of constriction and compression had been emptied through nature's opening into the bladder and through the incision made by me through the perineum. After the contents of the bowel below the constricted points had been discharged, nothing was left to pass through the incision that might keep it open, and it had healed at the bowel. We found that the knife entered the bowel in Douglas' cul de sac, making an opening in the gut from three-eighths to half inch in length. Neither the bladder, prostate gland, ureters, nor any blood vessels of any importance, were injured by the knife. This autopsy, as is often the case, revealed a different state of affairs to what was anticipated before death; for in this instance we expected to find a bifurcated rectum, one end entering the bladder and the other terminating in a cul de sac in the pelvic fossa, as fecal matter passed both from the penis and the perineal incision; but instead of this we found an unnatural flexure as stated above.

